# Porcine Model of Hemophilia A

**DOI:** 10.1371/journal.pone.0049450

**Published:** 2012-11-28

**Authors:** Yuji Kashiwakura, Jun Mimuro, Akira Onishi, Masaki Iwamoto, Seiji Madoiwa, Daiichiro Fuchimoto, Shunichi Suzuki, Misae Suzuki, Shoichiro Sembon, Akira Ishiwata, Atsushi Yasumoto, Asuka Sakata, Tsukasa Ohmori, Michiko Hashimoto, Satoko Yazaki, Yoichi Sakata

**Affiliations:** 1 Research Division of Cell and Molecular Medicine, Center for Molecular Medicine, Jichi Medical University, Shimotsuke, Tochigi-ken, Japan; 2 Transgenic Animal Research Center, National Institute of Agrobiological Sciences, Tsukuba, Ibaraki-ken, Japan; 3 Advanced Technology Development Team, Prime Tech Ltd., Tsuchiura, Ibaraki-ken, Japan; Emory University School of Medicine, United States of America

## Abstract

Hemophilia A is a common X chromosome-linked genetic bleeding disorder caused by abnormalities in the coagulation factor VIII gene (*F8*). Hemophilia A patients suffer from a bleeding diathesis, such as life-threatening bleeding in the brain and harmful bleeding in joints and muscles. Because it could potentially be cured by gene therapy, subhuman animal models have been sought. Current mouse hemophilia A models generated by gene targeting of the *F8* have difficulties to extrapolate human disease due to differences in the coagulation and immune systems between mice and humans. Here, we generated a porcine model of hemophilia A by nuclear transfer cloning from *F8*-targeted fibroblasts. The hemophilia A pigs showed a severe bleeding tendency upon birth, similar to human severe hemophiliacs, but in contrast to hemophilia A mice which rarely bleed under standard breed conditions. Infusion of human factor VIII was effective in stopping bleeding and reducing the bleeding frequency of a hemophilia A piglet but was blocked by the inhibitor against human factor VIII. These data suggest that the hemophilia A pig is a severe hemophilia A animal model for studying not only hemophilia A gene therapy but also the next generation recombinant coagulation factors, such as recombinant factor VIII variants with a slower clearance rate.

## Introduction

Hemophilia A is an inherited X-linked bleeding disorder caused by abnormalities in the coagulation factor VIII (FVIII) gene (*F8*). The genetic abnormalities result in FVIII deficiency, which in turn creates a bleeding diathesis, such as life-threatening bleeding in the brain and harmful bleeding in joints and muscles. The morbidity of hemophilia A is one in 5,000 male live births [1]. The current standard therapy for hemophilia A is intravenous injection of recombinant FVIII or monoclonal antibody-purified FVIII from plasma. Prophylactic administration of FVIII is effective in preventing harmful bleeding; however, hemophilia A patients are still not free from the risks of life-threatening intracranial and other harmful bleeding [1] [2]. In addition, severe hemophilia A patients develop antibody against FVIII (inhibitor) upon infusion of FVIII frequently [1].

Gene therapy, that enables sustained elevation of coagulation factor levels, will provide the next-generation therapy for hemophilia [1], [3], [4]. In fact, gene and cell therapy for hemophilia clinical trials were conducted. Compared with clinical trials of the gene therapy for hemophilia B [5], [6], gene and cell therapies for hemophilia A have had limited successes [7], [8]. Upcoming therapeutic alternatives for hemophilia A are FVIII variants with a slower clearance rate. Therapeutic factors, such as recombinant activated factor VII and plasma-derived activated prothrombin complex, are used for the treatment of hemophilia A patients with inhibitors, and the second generation therapeutic factors for hemophilia A patients with inhibitors are also currently under development. For studying next-generation therapeutics, good animal models are required. Hemophilia A mice generated by targeted ablation of mouse *F8* [9] have been the mainstay for assessment of hemophilia A gene therapy and evaluation of FVIII variants. However, there are significant species differences between mice and humans. For example, the half-life of human FVIII in the mouse circulation is very short, making it difficult to analyze the efficacy of human FVIII-expressing vectors for gene therapy or novel FVIII variants. As alternatives, there are natural hemophilia A dogs and hemophilia A sheep. Hemophilia A dogs have been used for gene therapy studies [10], [11], [12]. Hemophilia A sheep would be an alternative model [13]. There may be interspecies differences, such as body size, physiology, disease progression and chromosome structure homology, between these animal models and humans.

Pigs are excellent biomedical models of human diseases [14], [15]. The porcine blood coagulation system is very similar to that in humans, because of the high homology between the coagulation factor amino acid sequences [16], [17], [18]. In addition, porcine FVIII has been used to treat hemophilia A patients with FVIII inhibitors [19], [20], [21]. Therefore, the hemophilia A pig could be a good animal model to study the next-generation therapeutics for hemophilia A. Moreover, a miniature pig strain exists, and thus, cloned pigs could be downsized to an adequate size, approximately 20–30 kg in weight. For these reasons, we decided to generate hemophilia A pigs by cloning technology.

## Results

**Figure 1:**
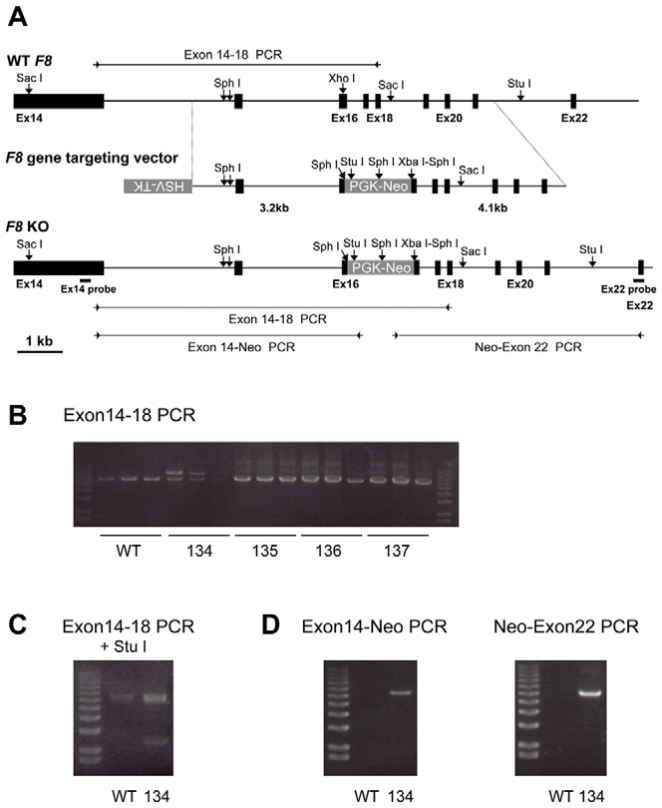
*F8* targeting of porcine fetal fibroblasts (PEF). (**A**) Schematic diagram of part of porcine *F8*, the positions of the restriction endonuclease sites, the *F8* targeting vector structure, and the targeted *F8* (*F8* KO) allele are shown. The neomycin-resistance gene (PGK-neo) was inserted in the exon 16 DNA fragment with deletion of a part of exon 16 and was flanked by two *F8* DNA fragments (5′ arm: 3.2 kb; 3′ arm: 4.1 kb) in *F8* targeting vector. The positions of PCR primers (arrowheads), expected amplified DNA fragments (bars), and restriction endonuclease sites used for the Southern blot analysis are indicated in the schema for *F8* KO. (**B**) *F8* exon 14–18 PCR on genomic DNA from non-transfected PEF (WT), PEF colony 134 (134), and three other PEF colonies (135–137) was shown. (**C**) The *F8* exon 14–18 PCR products were treated with *Stu* I and analyzed by agarose gel electrophoresis. (**D**) PCR analyses with two sets of primer pairs for exon 14 and the neomycin resistance gene and for the neomycin resistance gene and exon 22 were shown.

**Figure 2:**
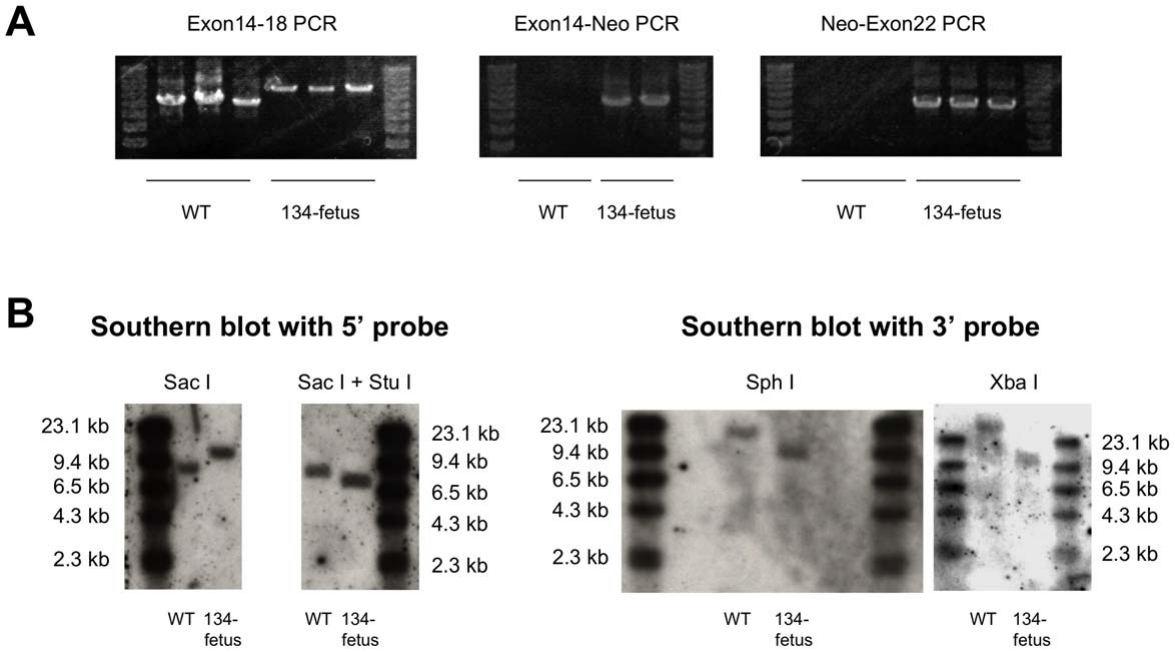
*F8* targeting and genetic analysis of the colony 134-derived fetus. PCR analysis of genomic DNA of 134-fetus was shown. (**A**) Two or three independent PCR reactions were carried out for detection of recombination in *F8* of 134-fetus. (**B**) Southern blotting with a 5′ exon 14 probe (on *Sac* I− or *Sac* I + *Stu* I-digested DNA) and with a 3′ exon 22 probe (on *Sph* I− or *Xba* I-digested DNA) showed correct targeting of the *F8* in 134-fetus.

Firstly, we constructed a *F8* targeting vector (Figure 1**A**) and targeted *F8* in male porcine embryonic fibroblasts (PEF) with the *F8*-targeting vector as shown in Figure 1. The DNA fragment amplified from the non-transfected PEF DNA migrated at 6.5 kb on agarose gel electrophoresis, whereas two DNA fragments, migrating at 6.5 kb and 8.3 kb, were amplified from PEF colony 134. The 8.3 kb DNA was not amplified from genomic DNA of PEF colonies 135–137. The 8.3 kb fragment PCR-amplified from PEF colony 134 was cleaved into a 2.4-kb fragment and a 5.9-kb fragment by *Stu* I, whereas the PCR-amplified 6.5-kb DNA fragment was not susceptible to *Stu* I digestion. This supports that the PCR-amplified 8.3-kb fragment is derived from the *F8*-targeted genome because a *Stu* I recognition sequence present in the neo-resistant gene but not in the PCR amplified DNA fragment from the wild-type *F8*. The expected DNA fragments were amplified by PCR with Neo primers from genomic DNA from PEF colony 134, but not from wild-type genomic DNA (WT). PCR analysis of genomic DNA with three primer sets revealed a recombination event in *F8* of a colony, 134 (PEF-134). PEF-134 nuclei were then injected into enucleated oocytes. After an electrical pulse, the oocytes were transplanted into the oviduct of a female pig [22], [23]. Transplantation of nucleus-transferred oocytes to the oviducts of female pigs was repeated four times. Three months later, a fetus was obtained by induced abortion. Dermal fibroblasts from this PEF-134-derived fetus (134-fetus) were isolated and cultured, and genomic DNA was isolated for analysis by PCR and by Southern blotting (Figure 2). The PCR amplified wild-type (WT) *F8* exon 14–18 fragment migrated at 6.5 kb, whereas the 8.3-kb targeted DNA fragment was amplified solely from 134-fetus fibroblast DNA. PCR-amplified DNA fragments using an *F8* exonic primer and a Neo primer were obtained only from 134-fetus DNA. The PCRs demonstrated insertion of the neomycin-resistance gene in *F8* (Figure 2**A**). Southern blotting showed that the 5′ probe hybridized with the 8.1 kb DNA fragment of Sac I-digested wild-type DNA while the 5′ probe hybridized with 9.9 kb DNA fragment of Sac I-digested 134-fetus DNA. Southern blotting with the 3′ probe confirmed recombination in the *F8* gene in the 134-fetus genome because a Sph I recognition sequence and a Xba I recognition sequence located in the 3′ end of the Neo resistant gene of the targeted allele (Figure 2**B**). Therefore, five transfers of fetal fibroblast nuclei to oocytes followed by oocyte transplantation were performed. Four females became pregnant and each produced a full-term delivery.

**Figure 3:**
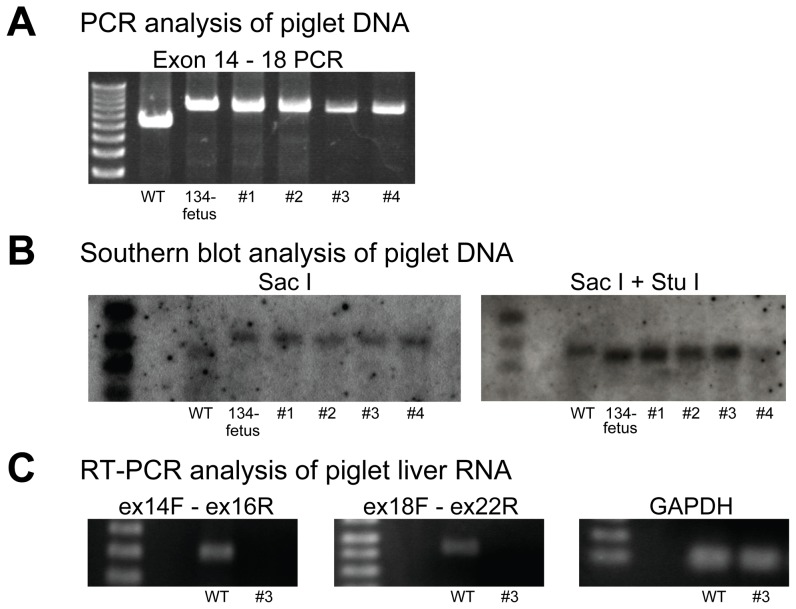
Analysis of the *F8* in cloned piglets. (**A**) PCR analysis of genomic DNA of piglet DNA was shown. Genomic DNA of wild-type, 134-fetus, piglet #1, piglet #2, piglet #3, and piglet #4 was subjected to PCR analysis with primers Exon 14 sF and Exon 18 sR as in Figure 1. The 8.3 kb exon 14–18 band was amplified from the 134-fetus DNA and the cloned piglet DNA. (**B**) Southern blotting with a 5′ exon 14 probe (on *Sac* I− or *Sac* I + *Stu* I-digested DNA) showed the same mobility shifts of the bands as those in Figure 2**B** and confirmed the insertion of the Neo resistant gene in *F8* of the cloned piglets. (**C**) RT-PCR analysis of piglet liver RNA was shown. Two independent PCRs (exons 14–16 and exons 18–22) revealed the absence of FVIII mRNA from the liver of cloned piglet #3. Control GAPDH mRNA was detected in the liver RNA of piglet #3 as in the wild type (WT).

Four live offspring were obtained and PCR analysis and Southern blotting were carried out. As shown in Figure 3**A**, the 8.3 kb DNA fragments were PCR amplified from piglets DNA as same as that of 134-fetus (Figure 2). Similarly, Southern blotting of Sac I-treated and *Sac* I and *Stu* I-treated DNA of the piglets with the 5′ probe confirmed the recombination of F8 of piglets and showed that each piglet had a single copy of the targeted *F8* (Figure 3, **A** & **B**). RT-PCR analysis revealed that FVIII mRNA was not detected in the liver of piglet #3 (Figure 3**C**). Analysis of the blood of piglets #3 and #4 confirmed that the FVIII level was severely decreased to less than 1%, using an activated partial thromboplastin time (APTT)-based coagulation assay for human FVIII (Table 1). Other coagulation factors were moderately decreased (Table 1). The levels of albumin and cholinesterase of these piglet blood were also measured as the references to study whether the decreased level of coagulation factors II, V, VII, IX, and X were specific or not. The albumin levels of piglet #3 and #4 were decreased significantly compared with the wild type piglets. However, the cholinesterase activities of piglets #3 and #4 were not decreased. The data suggested that synthesis of some proteins in the liver of the cloned piglets was altered. The precise mechanism of the moderately decreased levels of coagulation factors II, V, VII, IX, and X, and albumin was not elucidated in this study.

**Table 1:** Coagulation factor activity of piglets #3 and #4.

Coagulation factor	Wild type (n = 4)	Piglet #3	Piglet #4
Fibrinogen (µmol/L)	2.67±1.39	1.56	ND
Factor II (%)	75.7±3.9	53	47
Factor V (%)	>200	118	168
Factor VII (%)	68.5±3.4	19	19
Factor VIII (%)	>200	1>	1>
Factor IX (%)	>200	96	69
Factor X (%)	134±7.0	72	64

	Wild type (n = 4)	Piglet #3	Piglet #4
von Willebrand Factor (%)	174.7±25.9	124	251
Albumin (g/dL)	2.8±0.08	1.0	1.9
Cholinesterase (IU/L)	3.75±1.50	15	3

The coagulation factor levels of piglet #3 and #4 are shown with the control coagulation factor levels of wild-type piglets. Each coagulation factor activity was calculated from the standard curve generated with normal human plasma and expressed as the percentage of the respective coagulation factor activity in normal human plasma.

ND: not determined.

**Figure 4:**
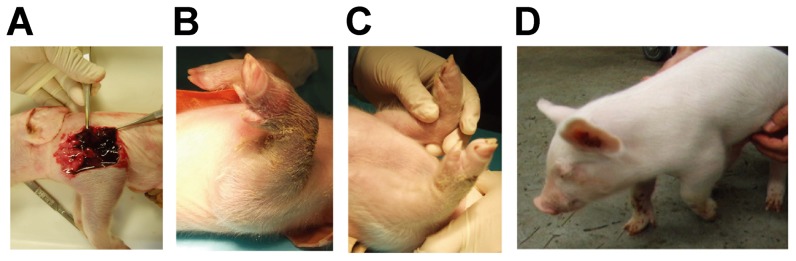
The bleeding phenotype of cloned *F8* KO piglets. (**A**) A part of macroscopic picture of cloned piglet #1, which died by day 2 after birth is shown. Ecchymosis was seen in the cheek, the forelimb, and the hind limb (not shown). Pathological examination revealed hematomas in these areas of piglet #1. (**B**) Forelimb of cloned piglet #4 on day 1 after delivery was shown. Ecchymosis had been seen in the left forelimb of cloned piglet #4 since delivery. (**C**) On day 5 after administration of human FVIII (150 U/kg), the bleeding in the left forelimb was not observed. Macroscopic picture of cloned piglet #4 on day 28 after birth showed that the left forelimb was swollen because of the repeated bleeding (**D**), causing the piglet to limp (also see video 1).

Two of the piglets (#1 and #2) found dead the next day (day 2) after delivery. The cause of death of these two piglets was not certain. Early deaths of cloned piglets after birth are not uncommon as described [24], [25]. Accidental bleeding might affect the condition of piglet #1 since large hematomas were observed in piglet #1 (Figure 4). Massive traumatic intramuscular bleeding was thought to affect the death of piglet #3 on day 3 because the general condition of piglet #3 became severe immediately after the bleeding took place and piglet #3 died. Piglet #4 was born with bleeding in the left forelimb, thus, human FVIII concentrate (150 U/kg) was injected intravenously on day 2 after delivery, which cured the bleeding in the limb (Figure 4). However, because this piglet still showed a bleeding in the limbs and the tongue, which was cured with human FVIII infusion, it was given a prophylactic infusion of human FVIII (150 U/kg) twice a week, which was effective in reducing the bleeding frequency. The human FVIII activity at 12.1% (average of two points; day 10 and day 23 after birth) was detected in the piglet #4 plasma obtained two days after the injection. However, spontaneous bleeding still occurred in piglet #4, in particular repeated bleeding in the left forelimb, causing limping (Figure 4 and video S1). Piglet #4 died due to gastric bleeding from a gastric ulcer on day 38 after birth. Inhibitor (856 BU/mL) against human FVIII was detected in the plasma obtained on the day when piglet #4 died. The development of inhibitor might explain why human FVIII injected two days before was not effective to reduce bleeding from the gastric ulcer.

## Discussion

Advances in cloning technology have allowed us to generate genetically modified animals [22], [26], [27]. Among these, a few gene-targeted pigs have been reported, such as cystic fibrosis pigs [28] and heterozygous fumarylacetoacetate hydrolase deficient pigs [29]as a disease model, and α1, 3-galactosyltransferase gene-knockout (KO) pigs [30] for organ transplantation [30], [31]. Considering the limitations in studying human disease in murine models, gene-targeted pigs are thought to be preferred for studying human diseases and for translational research. We explore the possibility of *F8* KO pigs (hemophilia A pigs) for studying the next generation therapy for hemophilia A in the current study. The genotype of cloned pigs showed the proper recombination in the *F8* of the pigs and the blood coagulation factor levels of cloned pigs confirmed severe FVIII deficiency. The precise mechanism of moderately decreased other coagulation factor levels in piglets #3 and #4 was not elucidated yet, these changes may not be specific to the coagulation factors since the level of albumin was decreased but the cholinesterase level was not decreased (both albumin and cholinesterase are synthesized in the liver). One possible mechanism of the changes could be the epigenetic effect genome DNA methylation and histone acetylation, which alter gene expression in cloned pigs [24], [32], [33], [34]. Hemophilia A pigs generated by the nuclear transfer technology did show a severe bleeding phenotype that is in contrast to *F8* KO mice that rarely exhibit spontaneous bleeding into the muscles and joints under standard breed conditions [9]. Therefore, hemophilia A pig can be used to evaluate an efficacy of novel therapy such as gene therapy for hemophilia A in a standard breed condition. Moreover, prophylactic infusion of human FVIII was effective in reducing bleeding in *F8* KO piglet #4 thought its therapeutic effect was not perfect. This suggests that the *F8* KO pig is a subhuman animal model of severe hemophilia A for the study of upcoming therapeutic factors, such novel FVIII variants. Piglet #4 died because of bleeding from a gastric ulcer. Since inhibitor against human FVIII was detected in the plasma sample obtained on the day when piglet #4 died, the therapeutic effect of human FVIII no longer existed at the time, resulting in severe bleeding from the gastric ulcer. It is possible that *F8* KO pigs might develop antibodies against porcine FVIII as against human FVIII. The possibility of the use of *F8* KO pigs as a model for studying immune tolerance induction therapy for FVIII inhibitor remains to be studied.

## Methods

### Construction of the F8 targeting vector

Porcine genomic DNA was isolated from porcine embryonic fibroblasts (LW; Landrace – Large White, ED65). The *F8* targeting vector was constructed by inserting two genomic DNA fragments into the plasmid vector pHSV-TK/PGK-Neo. The *F8* targeting vector was designed by referring to the *F8* exon 16 gene-targeting vector used to generate hemophilia A mice [9]. *F8* DNA fragments from exons 14–22 were isolated by PCR using primers (Table S1) based on the *Sus scrofa* coagulation factor VIII mRNA sequence (accession number: NM_214167) and sequenced. The two homologous arms of the gene-targeting vector were generated by reference to this sequence. The 5′ DNA fragment spanning intron 15 to exon 21 of *F8* was PCR-amplified, digested with *Xho* I to generate an 11-nucleotide deletion of exon 16, and inserted into pHSV-TK/PGK-Neo. The 3′ DNA fragment was PCR-amplified from exon 16 to intron 21, and cloned into pHSV-TK/PGK-Neo containing the 5′ *F8* DNA fragment. The herpes simplex virus thymidine kinase gene was located in the opposite orientation on the 5′ end of the 5′ arm. The targeting vector was linearized with *Not* I before transfection.

### Isolation of porcine embryonic fibroblasts and isolation of F8-targeted cells

Porcine embryonic fibroblasts (PEF) were isolated from a male fetus of the LW strain as described [22]. PEFs (1×10^7^ cells) were transfected with the *F8* targeting vector by electroporation (Gene Pulser II; Bio-Rad, Hercules, CA) at 278 V and 950 µF. After transfection, cells were cultured in Dulbecco's modified Eagle's medium with low-glucose (Invitrogen, Carlsbad, CA) containing 10% fetal bovine serum. After 48 h incubation, cells were selected with 800 µg/ml G418 (Nacalai Tesque, Inc., Kyoto, Japan) and 2 µM gancyclovir (Tanabe-Mitsubishi Pharma, Tokyo, Japan). On the eighth day following selection, G418-resistant colonies had grown. Cells from these colonies were grown in 24-well plates (Corning) in medium containing 4 ng/ml bFGF, and expanded for genomic DNA extraction and storage. DNA isolated from three wells of each colony was analyzed by three independent PCR reactions for recombination in the porcine *F8* (Table S1).

### Southern blotting

Southern blotting for the *F8* recombination was performed by the standard procedure. Digoxigenin (DIG)-labeled 5′ and 3′ probes were generated by PCR (497 bp from exon 14 and 469 bp from exon 22, respectively) (Table S1). Signals were visualized using a DIG detection module (anti-DIG-alkaline phosphatase and a CSPD) (Roche Diagnostics GmbH., Mannheim, Germany).

### RT-PCR of porcine FVIII mRNA

Total RNA was isolated from piglet liver using an RNeasy Mini kit (Invitrogen), converted to cDNA and PCR amplified using the SuperScript One-Step RT-PCR System (Invitrogen) with primer pairs specific for FVIII mRNA (Table S1) [35], [36].

### Nuclear transfer and transplantation of manipulated embryos to recipients

Production of clone piglets by nuclear transfer was performed as described previously [22], [23]. In brief, metaphase II oocytes were enucleated by gentle aspiration of the first polar body and adjacent cytoplasm using a beveled pipette (25 to 30 µm) in PZM3 medium containing 5.0 µg/ml cytochalasin B. Enucleated oocytes were washed in PZM3 medium lacking cytochalasin B and nuclei of the *F8*-targeted cells introduced by direct intracytoplasmic injection using a piezo-actuated micromanipulator (Prime Tech., Tsuchiura, Japan). Oocytes were then stimulated with a direct current pulse of 1.5 kV/cm for 100 µS using a somatic hybridizer (SSH-10, Shimadzu, Kyoto, Japan) and transferred to PZM3 supplemented with cytochalasin B to prevent extrusion of a pseudo-second polar body. The nuclear transferred oocytes were then cultured in PZM3 medium in an atmosphere of 5% CO_2_, 5% O_2_ and 90% air at 38.5°C for 2 days until reaching the two-to-eight-cell stage. Cleaved embryos were transferred to the oviducts (200 embryos per recipient) of an anesthetized pseudopregnant surrogate mother (matured LWD; a Landrace×Large White×Duroc triple cross). Following embryo transfer, mother pigs were observed daily to confirm pregnancy by checking estrus. Farrowing was synchronized by injection of the prostaglandin F2α analog, (1)-cloprostenol (Planate, Osaka, Japan) on day 113–116 of gestation.

### Coagulation factor activity measurement

Activities of porcine coagulation factors were measured at a clinical laboratory (SRL, Tokyo, Japan) by the standard clotting time method with respective coagulation factor-deficient human plasma. Normal human plasma was used as the standard for each test. The coagulation factor activity in piglet plasma was expressed as the percentage of the coagulation factor activity in normal pooled plasma, except for fibrinogen. The fibrinogen concentration was determined by the thrombin time method. von Willebrand factor levels in pig plasma were measured with an enzyme immunoassay with latex particle conjugated antibody (performed at SRL, Tokyo, Japan) since the von Willebrand factor activity (Ristocetin cofactor activity) in pig plasma was unable to be measured with human platelets. The von Willebrand factor antigen levels in pig plasma were expressed as percentages of the normal human plasma. An inhibitor assay for human FVIII was performed as described [36].

### Blood chemistry analysis

The levels of albumin and choline esterase of piglet blood were measured at the Nagahama Life-science Laboratory of Oriental East Co. Ltd (Hagahama, Shiga-ken, Japan). Choline esterase activities of blood samples were measured with p-hydroxy benzoyl choline iodide as the substrate [37].

### Animal experiments

All the animal experiments and surgical procedures were carried out in accordance with guidelines approved by the Institutional Animal Care and Concern Committees of Jichi Medical University and the National Institute of Agrobiological Sciences. Protocols for the use of animals in this study were approved by the review boards of Animal Care Committees of Jichi Medical University and the National Institute of Agrobiological Sciences. Wild type pigs used in this study were bred under a standard condition according to the institutional guideline of Animal Care Committee of National Institute of Agrobiological Sciences. After delivery, cloned F8KO pigs were separated from mother pigs and each cloned F8KO pig was bred by artificial suckling in a cage with protection of soft buffers to avoid traumas. All the experimental procedures including injection of FVIII were carried out under inhalation anesthesia with isoflurane and monitoring of body temperature. The endpoint of this study was to generate F8KO pigs and analyze the genotype and the phenotype of the F8KO pig precisely to investigate whether the F8KO pig can be a subhuman model of severe hemophilia A.

## Supporting Information

Table S1 **Sequences of primers used in this study.**
(DOC)

Video S1 **Piglet #4 (day 28 after birth) to limp in the left forelimb.**
(MOV)
