# Supplementary material for: Porcine Model of Hemophilia A
Source: PLoS One. 2012 Nov 28;7(11):e49450. doi: 10.1371/journal.pone.0049450 (PMC3509096; doi:10.1371/journal.pone.0049450)
Supplement: Table S1 — Sequences of primers used in this study. (DOC) [file pone.0049450.s001.doc]

Supplementary data:

Porcine Model of Hemophilia A

Yuji Kashiwakura*^1^, Jun Mimuro*^1^§, Akira Onishi*^2^, Masaki Iwamoto*^2,3^, Seiji Madoiwa^1^, Daiichiro Fuchimoto^2^, Shunichi Suzuki^2^, Misae Suzuki^2^, Shoichiro Sembon^2^, Akira Ishiwata^1^, Atsushi Yasumoto^1^, Asuka Sakata^1^, Tsukasa Ohmori^1^, Michiko Hashimoto^3^, Satoko Yazaki^3^, Yoichi Sakata^1^§

^1^Research Division of Cell and Molecular Medicine, Center for Molecular Medicine,

Jichi Medical University, Tochigi-ken 329-0498, Japan.

^2^Transgenic Animal Research Center, National Institute of Agrobiological Sciences, Tsukuba, Ibaraki-ken 305-8602, Japan.

^3^Prime Tech Ltd., Tsuchiura, Ibaraki-ken, 300-0841, Japan

*These authors contributed equally to this work.

§Correspondence should be addressed to Jun Mimuro (mimuro-j@jichi.ac.jp) or Yoichi Sakata (yoisaka@jichi.ac.jp)

**Supplementary Table**

**Table S1. Primer sequences**

**Primers for construction of the gene-targeting vector**

Exon 14F; 5’-CAGGACTCGAGAAGAGAACCCGACACTATTTCATTGCTGC-3’

Intron 14F: 5’-CCGCTCGAGGAGTTCCTGCTATGGCACG-3’

Exon 21R: 5’-CTCAGATTGATCCGGAATAATGAAGTCTGGCCAGC-3’

Exon 16F: 5’-CTCTAGATCCGGATGATCAGGAGCAAGGGGC-3’

Intron 21R: 5’-GCCTCTAGATCTGCGACCTACACCACAGCTC-3’

Exon 22R: 5’-CTCTAGAGGTAACTCTGCCAGTTCCTCCCGTCAAGAC-3’

**Primers for PCR Screening for *F8* recombination**

Exon 14 sF: 5’-GCTTTATTAACTGAGAATAGGGCATCTGC-3’

Exon 18 sR: 5’-GCCAGGGAGTGTATCCATCACATAG-3’

Neo sR: 5’-CTTCCCGCTTCAGTGACAACGTCGAGCACAG-3’

Neo sF: 5’-CGCCTTCTTGACGAGTTCTTCTG-3’

Exon 22 sR: 5’-TAAGGTGCCCGTGGAATTCCCTC-3’

**Primers for RT-PCR detection of porcine *F8* mRNA**

Exon 14 F: 5’-CGACACTATTTCATTGCTGC-3’

Exon 16Δ R: 5’-CCGGATAAGAAATAAGGCTC-3’

Exon 18 F: 5’-GTACAATCTCTATCCGGGTG-3’

Exon 22 R: 5’-GGTAACTCTGCCAGTTCCT-3’

GAPDH F: 5’-AAGCTCATTTCCTCGTACGAC-3’

GAPDH R: 5’-GGAGGCCATGTGGACCAT-3’

**Primers for the DIG-labeled probes**

Exon 14 F: 5’-GAAATCGAACAGGTCCAGG-3’

Exon 14 R: 5’-CAGAAGCTTGGAGGGAGTC-3’

Intron 21 F: 5’-TACAGCGCTTCCTTACCAC-3’

Exon 22 R: 5’-TAACTCTGCCAGTTCCTCC-3’
